# A Case of Congenitally Corrected Transposition of the Great Arteries Discovered on Coronary Computed Tomography

**DOI:** 10.1155/2013/420213

**Published:** 2013-02-17

**Authors:** Adam T. Marler, Jennifer N. Slim, Travis Batts, James Watts, Ahmad M. Slim

**Affiliations:** Internal Medicine and Cardiology Service, San Antonio Military Medical Center, 3851 Roger Brooke Drive, Fort Sam Houston, TX 78234-6200, USA

## Abstract

Congenitally corrected transposition of the great arteries is a rare condition accounting for less than 1% of all congenital cardiac diseases. The fundamental nature of this condition involves a blend of atrioventricular as well as ventriculoarterial discordance. Congenitally corrected transposition of the great arteries is classically associated with three additional abnormalities, including ventricular septal defect, right ventricular outflow tract obstruction, and tricuspid valve abnormalities. Patients with this anomaly have been shown to exhibit reduced exercise tolerance as well as reduced health-related quality of life when compared to patients with normal cardiovascular anatomy. We present the case of a 33-year-old active duty lieutenant in the United States Air Force referred to the cardiology clinic for evaluation of valvular heart disease with subsequent discovery of congenitally corrected transposition of the great arteries on cardiac gated computed tomography.

## 1. Case Report

A 33-year-old male with a history of secundum atrial septal defect percutaneously ended in May 2008 referred for further evaluation of valvular heart disease. Transthoracic echocardiogram completed for a heart murmur was noted to be technically difficult with suboptimal image quality due to mesocardia. Official report from this examination detailed grossly normal left ventricular wall motion with moderate, posteriorly directed, eccentric mitral regurgitation, mild prolapse of the anterior mitral valve leaflet, mild aortic insufficiency, and an echo bright structure contiguous to the lateral aspect of the tricuspid annulus. The right ventricle and right atrium were not well visualized. 

**Figure 1:**
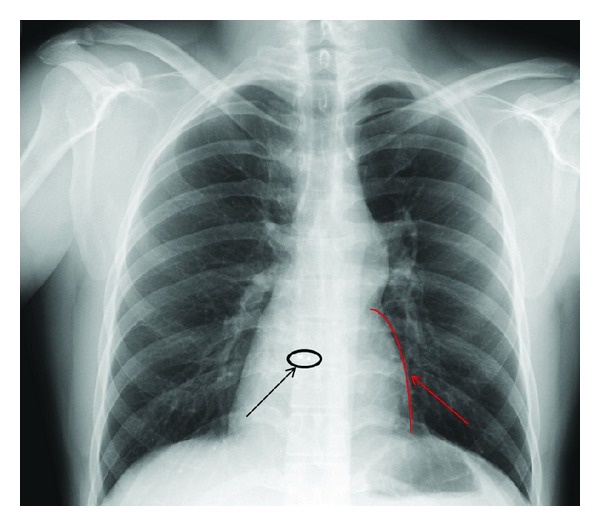
X-ray changes anticipated in ccTGA with right ventricular border outlined with red arrow and Amplatzer occluder device outlined with dark arrow.

At initial visit he reported feeling well overall, but he felt fatigued at 200–400 meters. Exercise stress testing was completed with the patient exercising 12 minutes 26 seconds on a standard Bruce protocol achieving a workload of 14.2 metabolic equivalents (METS) achieving 96% of maximal age-predicted heart rate with exercise. Exercise testing terminated due to fatigue with the patient reporting no chest pain during evaluation. No ischemic changes were noted on electrocardiogram during the study. Patient's chest X-ray was suggestive of possible RV on the left side of the chest with possible dextrocardia or ccTGA in the differential (Figure 1).

**Figure 2:**
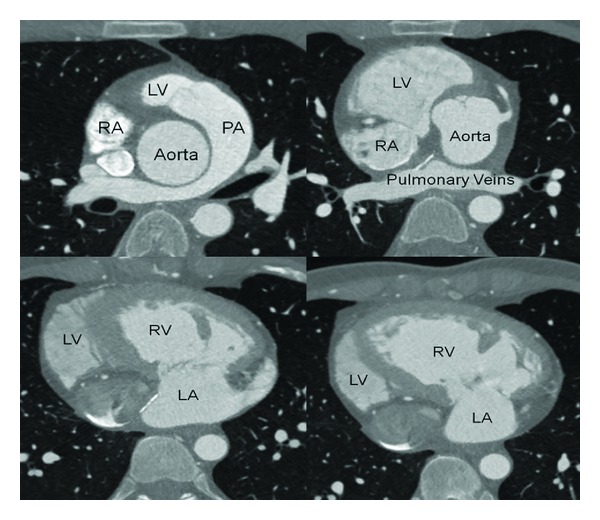
Anatomical confirmation of ccTGA on coronary CT (CTA).

Subsequently, the patient underwent imaging evaluation with cardiac computed tomography (CT) for further evaluation of cardiac structure. CT angiography of the coronary arteries revealed normal origin and course of all coronary vessels with no evidence of coronary artery disease. CT evaluation of cardiac structure showed the right ventricle receiving blood from the left atrium through the tricuspid valve and the left ventricle receiving blood from the right atrium through the mitral valve. The right ventricle was shown to eject blood into the aorta with the left ventricle pumping blood into the pulmonary artery. The right ventricle demonstrated concentric hypertrophy measuring 9.9 mm at the mid-interventricular septum. Findings were noted to be consistent with a congenitally corrected L-transposition of the great arteries (ccTGA) (Figure 2).

Following imaging evaluation, the patient was diagnosed with systemic hypertension and begun on hydrochlorothiazide for initial therapy. Antihypertensive therapy was changed from hydrochlorothiazide to lisinopril during a brief admission for atypical chest pain due to failure to reach treatment goals with the diuretic. Mandatory military Medical Evaluation Board (MEB) was initiated due to the presence of moderate valvular disease and, as part of this process, cardiopulmonary exercise testing was scheduled despite recent history of gated exercise testing and no worsening of daily function per patient report. Cardiopulmonary exercise testing was carried out using cycle ramp protocol beginning at 0 Watts and incrementally increasing to workload of 157 Watts. The patient exercised for 7 minutes 10 seconds with testing discontinued due to leg fatigue. Peak VO_2_ measured at 29.7 mL/kg/min representing 8.5 METS or 77% of maximum predicted. Anaerobic threshold occurred at 51% of measured VO_2_ max. Peak heart rate was 180 beats per minute with a blood pressure peak of 146/70. There was delayed heart rate recovery with noted heart rate of 150 beats per minute at three minutes of recovery. Peak respiratory rate was 42 per minute with VE of 68 L/min and VE/VCO_2_ slope of 29.9 and pulse oximeter reading of >95%. Overall capacity of the patient has dropped markedly as compared to the 14.2 METS achieved on a standard exercise stress test performed a year prior.

## 2. Discussion

Congenitally corrected transposition of the great arteries (ccTGA) accounts for less than 1% of all congenital cardiac anomalies [1]. Most patients with ccTGA have at least one additional cardiac abnormality consisting of pulmonary stenosis (41%), ventricular septal defects (37%), and atrial septal defects (19%) as well as Ebstein's anomaly of the tricuspid valve (7%) [2]. In our patient, atrial septal defect was detected and an occlude device was placed but yet the diagnosis of ccTGA was never contemplated till a chest X-ray was obtained followed by coronary CT for confirmation. The utility of coronary CT (CTA) is increasing in screening military service members for the workup of acute symptoms of chest pain; however, this case proves that screening CTA for military recruits with any concern of congenital disorder might be warranted with excellent accuracy when Transthoracic echocardiography proves to be limited.

In a retrospective study of 41 patients with ccTGA who had completed lung function testing and cardiopulmonary exercise testing, aerobic capacity in patients with ccTGA is severely diminished with VO_2_ max ranged from 11 to 22 mL/kg/min that corresponds to 30–50% of results achieved by healthy patients [3]. In the same retrospective study, systemic RV ejection fraction rose by only 2% in ccTGA patients, while pulmonary LV ejection fraction reduced by 2% with exertion. Patients on ACE inhibitors had significantly lower pulmonary LV ejection fraction at max exertion compared to those not on ACE inhibitors [3]. These findings might explain the acute reduction in exercise capacity in our patient immediately after initiation of ACI for blood pressure control.

## 3. Conclusion

Congenitally corrected transposition of the great arteries is a rare cardiac anomaly where many patients will remain asymptomatic for much of their lives. ccTGA patients have a reduced tolerance for exercise and have reported reduced health-related quality of life compared to a control population. As noted in this patient, ACE inhibitors may intensify exercise intolerance in these patients.
